# Characterization of biofilm-forming capacity and resistance to sanitizers of a range of *E. coli* O26 pathotypes from clinical cases and cattle in Australia

**DOI:** 10.1186/s12866-018-1182-z

**Published:** 2018-05-08

**Authors:** Salma A Lajhar, Jeremy Brownlie, Robert Barlow

**Affiliations:** 1 School of Environment and Science, Griffith University, Brisbane, QLD Australia; 2 Present address: CSIRO Agriculture and Food, 39 Kessels Rd, Coopers Plains, Brisbane, QLD 4108 Australia

**Keywords:** *E. coli* O26, Biofilm, Food sanitizers, Curli, Cellulose, Hydrophobicity, Polystyrene, Stainless steel, Pellicle, *mlrA*

## Abstract

**Background:**

The formation of biofilms and subsequent encasement of bacterial cells in a complex matrix can enhance resistance to antimicrobials and sterilizing agents making these organisms difficult to eradicate and control. The aim of this study was to evaluate and compare the capacity of 40 *E. coli* O26 isolates of enterohemorrhagic *E. coli* (EHEC, *n* = 27), potential EHEC (pEHEC, *n* = 3), atypical enteropathogenic *E. coli* (aEPEC, *n* = 8) and non-toxigenic *E. coli* (NTEC, *n* = 2) from human and cattle sources to form biofilms on different surfaces, and determine whether extracellular matrix (ECM) components (cellulose, curli), motility, prophage insertion in *mlrA* and cell surface hydrophobicity could influence biofilm formation. Finally, the influence of biofilm formation on the sensitivity of isolates to quaternary ammonium compounds (QACs; Profoam, Kwiksan 22) and peracetic acid-based sanitizer (Topactive Des.) for 2 min on polystyrene plate were also evaluated.

**Results:**

Biofilm production on one surface may not indicate biofilm formation on a different surface. Biofilm was formed by different pathotypes on polystyrene (70%), stainless steel (87.5%) and glass slides (95%), however only 50% demonstrated pellicle formation. EHEC isolates were significantly more likely to form a pellicle at the air-liquid interface and biofilms on polystyrene surface at 48 h than aEPEC. Strains that don’t produce ECM (curli or cellulose), harbor a prophage insertion in *mlrA,* and are non-motile have lower biofilm forming capacities than those isolates possessing combinations of these attributes. Hydrophobicity had no impact on biofilm formation. After 2 min exposure, none of the disinfectants tested were able to completely inactivate all cells within a biofilm regardless of pathotypes and the amount of biofilm formed.

**Conclusion:**

Pathotypes of *E. coli* O26 showed varying capacities to form biofilms, however, most EHEC strains had the capacity to form biofilm on all surfaces and at the air-liquid interface under the conditions used in this study. Biofilms provided a protective effect to *E. coli* O26 strains against the three sanitizers, previously shown to successfully control the growth of their planktonic counterparts. Whether the characteristics of biofilm forming and non-biofilm forming strains observed in this study reflect their attributes within the food and meat-processing environments is unknown. Further studies that represent the food and meat-processing environments are required.

**Electronic supplementary material:**

The online version of this article (10.1186/s12866-018-1182-z) contains supplementary material, which is available to authorized users.

## Background

Enterohaemorrhagic *E. coli* (EHEC) strains are foodborne pathogens that have been implicated in a number of outbreaks with symptoms ranging from diarrhoea to haemolytic uremic syndrome (HUS) which can lead to death. Although O157 is the most common serogroup associated with disease, a number of other serogroups such as O26, O45, O103, O111, O121 and O145 are now considered as major causes of foodborne illness worldwide [1–3]. Food producing animals, particularly cattle have been identified as a major reservoir of these pathogens and there have been several outbreaks attributed to the consumption of contaminated meat and milk products [4–8]. Furthermore, in the United States of America (USA), EHEC of serogroup O157:H7 and the additional six serogroups O26, O45, O103, O111, O121 and O145 are considered adulterants of both raw, non-intact beef products such as ground beef, veal patties, and beef patties mix, and intact beef cuts that are to be further processed into non-intact cuts such as manufacturing trimmings of meat remaining after steaks or roasts are removed [9, 10]. Although the number of sporadic and outbreak cases of EHEC disease in Australia remains low [11], EHEC are of economic importance as the Australian cattle industry is a significant exporter of red meat products. Investigating and controlling these pathogens is crucial in maintaining access to markets such as the USA and any others that regulate for the presence of EHEC. Among EHEC, *E. coli* O26 is one of the most common non-O157 serogroups associated with serious foodborne outbreaks worldwide [8, 11–15] with a number of food outbreaks linked to consumption of beef products and cattle dairy products [4, 5, 8, 14, 16, 17]. In Australia, EHEC, serogroup O26, can be isolated from human clinical cases [11] and beef and dairy cattle [18, 19] albeit the prevalence in cattle populations and annual notification rate of EHEC O26-associated disease appears to be low when compared to other countries [11, 19]. Nonetheless, as EHEC illness can lead to life threating disease such as HUS, presence of this organism represents a growing concern to the public health authorities and Australian red meat exporters and subsequently there is a need to understand how these organisms persist and transfer into farm-to-fork production chain.

Foodborne pathogens such as *E. coli* use a range of strategies to survive and persist in the environment. It has been shown that various *E. coli* serogroups including *E. coli* O26 have the capacity to form biofilms [20–22]. The formation of biofilms and subsequent encasement of bacterial cells in a complex matrix can enhance resistance to antimicrobials and sterilizing agents making these organisms difficult to eradicate and control [21–23]. Several factors have been demonstrated to affect biofilm formation [20–22, 24, 25] including expression of extracellular matrix components (ECM; curli and/or cellulose), temperature, hydrophobicity, surface charge, surface structures and material properties. In addition, recent studies [25, 26] suggested that a prophage insertion in a MerR-like regulator; *mlrA* (renamed from *yehV*) can act as a barrier that limits curli expression and consequently biofilm formation. The sigma factors RpoS and RpoD participate in the transcription of *mlrA* [27] which is induced in the stationary phase. The importance of *mlrA* is attributed to its role in regulating the expression of the DNA-binding transcription factor (*csgD*) which is in turn is required for the expression of curli and cellulose [27].

The role of biofilm formation in human infection and contamination of food products has been well investigated [28, 29]. It has been suggested that biofilms in food-producing facilities act as a source of bacteria that may contaminate food products causing food spoilage, human infections and severe illness [28, 29]. In addition, dissemination of Stx-encoding bacteriophages can occur within biofilms and potentially enable the emergence of new *E. coli* pathotypes [30].

A number of studies have investigated the biofilm forming capacity of non-O157 serogroups including EHEC O26, and the effectiveness of disinfectant interventions in restricting the growth of biofilms [21, 22]. Whilst these studies gave insight into the protective effect of biofilms, it is important to understand whether the survival of pathogens to disinfectants differs depending on whether the cells are in a planktonic or biofilm state. Furthermore, it is of greatest relevance to the Australian food industry if a study utilises disinfectants that are typically used in industry and isolates that have been isolated from Australian cattle or human clinical cases. The aim of this study was to evaluate and compare the capacity of *E. coli* O26 isolates from human clinical and cattle to form biofilm on different surfaces, and determine the association of biofilm with pathotypes, ECM components (cellulose, curli), motility, prophage insertion in *mlrA* and bacterial adhesion to hydrocarbons. Finally, the influence of biofilms on an isolates sensitivity toward the three sanitizers previously shown [31] to be effective against their planktonic counterparts was also investigated.

## Methods

### Bacterial isolates

A total of 40 Australian clinical and cattle sourced *E. coli* O26 strains previously shown to represent the genetic diversity of Australian isolates were selected [31]. The strains were selected from a collection of 88 isolates based on their initial characterization by pulsed-field gel electrophoresis (PFGE), PCR for *stx, eae, ehx, bfp, ecf* and a single nucleotide polymorphism within *rmlA* along with their survival capabilities to disinfectants, acid approved for use in Australian food industry and antimicrobial susceptibility [31]. Based on the presence or absence of *stx1*, *eae*, *ehx*, *ecf, bfp, rmlA* SNP isolates were assigned into four pathotypes [31]. Cattle isolates were comprised of four pathotypes: enterohemorrhagic *E. coli* (EHEC; *n* = 27), atypical enteropathogenic *E. coli* (aEPEC; *n* = 8), non-toxigenic *E. coli* (NTEC; *n* = 2) and potential EHEC (pEHEC; *n* = 3; representing 30 distinguishable PFGE profiles while human clinical isolates were all EHEC O26 (*n* = 10) and represented 10 distinguishable PFGE profiles. In addition, all isolates had wild type RpoS except two human clinical isolates (EC4164QH7 and EC4165QH8) which had mutation in RpoS (data not published).

### Detection of curli and cellulose on Congo Red Indicator (CRI) agar

Curli and cellulose production was assessed on Congo Red Indicator (CRI) agar containing low salt (5 g/L) Luria-Bertani broth (LS-LB) supplemented with 40 mg/L of Congo red (Sigma-Aldrich, USA) and 20 mg/L brilliant blue (Sigma-Aldrich, USA). Bacterial isolates were initially cultured on LB agar (Oxoid, UK) and a single colony was inoculated into LS-LB broth and incubated statically for 18 h at 37 °C. An aliquot of 30 μl was spotted on CRI agar and incubated for 24 h at 37 °C, 48 h at 30 °C or 72 h at 25 °C. Expression of ECM components was determined based on colony morphology (RDAR: red colony, expresses curli fimbriae and cellulose, PDAR: pink colony, expresses cellulose, BDAR: brown colony, expresses curli fimbriae and SAW: no expression of curli fimbriae or cellulose morphotype) [32].

### Motility

Isolates were tested for motility in standard motility agar containing 3 g/L agar. Motility was investigated after 48 h at 25 °C. Non motile isolates were re-examined each 24 h for up to 7 days. Isolates that did not show motility in 3 g/L agar were subsequently passaged up to three times in fresh low-percentage-motility media containing 2 g/L agar in an effort to induce motility. Each isolate was examined in triplicate.

### Prophage insertion in *mlrA* (*yehV)*

To identify whether a prophage is inserted in the *mlrA*, all isolates were screened by PCR using each of primer sets *yehV*-attB (A: AAGTGGCGTTGCTTTGTGAT and B: ACAGATGTGTGGT GAGTGTCTG) and *yehV*-attL, (F: CACCGGAAGGACAATTCATC, B: AACAGATG TGTGGTGAGTGTCTG) [33]. The PCR amplification reaction contained 2 μl of boil cell lysate and 23 μl of master mix that consisted of 10X Dream Taq™ Buffer (Thermo Fisher Scientific, Australia), 250 mM dNTPs (Thermo Fisher Scientific, Australia), 0.02 mg/ml bovine serum Albumin (Sigma-Aldrich, USA), 12.5 pmol forward and reverse primer (GeneWorks, Australia) and 1.25 U Taq DNA polymerase (GeneWorks, Australia). The PCR conditions used were 94 °C for 5 min, followed by 30 s at 94 °C, 30 s at 62 °C, and 60 s at 72 °C for 30 cycles and finally 72 °C for 5 min. Amplified PCR products were analysed by gel electrophoresis, stained with ethidium bromide and the bands were visualised with UV transilluminator. Using the F/B primer pair, amplification of a 702 bp DNA product is expected when a prophage is inserted in the *mlrA* loci (interrupted *mlrA* loci); when no prophage inserted in *mlrA* (intact loci), a 340 bp products is expected to be amplified using primers A/B.

### Cell surface hydrophobicity

Cell surface hydrophobicity was measured using the bacterial adhesion to hydrocarbons (BATH) assay as described previously using xylene (Reagent Plus, 99%; Sigma-Aldrich, USA) [34] and hexadecane (Reagent Plus, 99%; Sigma-Aldrich, USA) [20]. The test was performed at 25 °C (48 h incubation) and 37 °C (24 h incubation). Following incubation, a 1 ml aliquot of the lower aqueous layer was gently aspirated and the OD_600_ was measured. All OD measurements were determined using Novaspec II spectrophotometer (Pharmacia Biotech Ltd., UK). The percentage of bound cells to hydrocarbon for each isolate was calculated according to the following formula: [(OD_600_ untreated bacterial cells - OD_600_ aqueous phase)/ OD_600_ untreated bacterial cells]*100.

### Biofilm formation on polystyrene microtiter plates

Assessment of biofilm formation on polystyrene plates at 24, 48 or 72 h at 25 °C without shaking was performed as described previously [22]. In brief, cultures were prepared by initially inoculating a single colony into LS-LB broth and incubating for 16–18 h at 37 °C with shaking at 150 rpm to reach a cell concentration of 8 log_10_ CFU/ml. The resulting enrichment was 100-fold diluted in sterile LS-LB and added to 96-well flat-bottom polystyrene plates (Sarstedt, USA) at 200 μl per well. Plates were incubated for 24, 48 or 72 h at 25 °C without shaking. Following incubation, the bacterial suspension was removed and plates were washed in triplicate with 270 μl sterilized phosphate-buffered saline (PBS; pH 7.2) to remove unattached or loosely attached cells. The plates were then air dried and stained with 100 μl per well of 0.1% crystal violet (CV) for 20 min. The plates were washed three times with PBS to remove excess stain, air dried and then 100 μl per well of 85% ethanol was added to each well to dissolve CV. Absorbance of the samples (As) were measured at optical density (OD_570_) using a microplate reader (EnSpire® Multimode Plate Reader-PerkinElmer, USA) and the degree of biofilm formation was assessed by subtracting the mean of parallel assays from the average absorbance of the negative control (Ac). At least two biological replicates were performed, each containing six technical replicates well per isolate. Based on the OD produced by bacterial biofilms at 570 nm, isolates were classified into these categories as previously described [35]: As ≤ Ac = no biofilm producer, Ac < As ≤ (2 × Ac) = low biofilm producer, (2 × Ac) < As ≤ (4 × Ac) = moderate biofilm producer and (4 × Ac) < As = strong biofilm producer. Sterile LB broth was used as a negative control and *Salmonella typhimurium* strain ATCC 14028 was used as a positive control in all biofilm experiments as it is known to produce RDAR at 28 °C but SAW at 37 °C [36].

### Biofilm formation on stainless steel and glass slides

Stainless steel coupons (0.9 mm thickness, size 50 × 20 mm) were prepared by being soaked in acetone for 30 min to remove contaminants and rinsed in water prior to soaking in 1 N NaOH for one hr. After soaking in 1 N NaOH, the stainless steel coupons were rinsed with distilled water and sterilized by autoclaving. No pre-treatment of glass slides, other than autoclaving, was performed and slides were used as manufactured. One ml aliquots of overnight culture (approximately 8 log_10_ CFU/ml) were inoculated into 50 ml sterile Röhre tubes (Sarstedt, Germany) containing 9 ml of LS-LB. A sterile glass slide (76 by 26 mm; Menzel GmbH+CoKG, Braunschweig, Germany) or a sterile stainless steel coupon was placed in each tube and only partially submerged in the broth to have an atmospheric interface with the liquid. The tubes were incubated at 25 °C for 72 h without shaking. After incubation, the slides/coupons were washed with water and then transferred to a test tube with 1% CV solution for staining of the biofilm for 20 min. Excess CV solution was rinsed from the slides/coupons using water. LB broth was used as a negative control. Stainless steel coupons and glass slides were then examined visually and given scores ranging from 0 (no visible biofilm) to 3 (thick biofilm at the air-liquid interface) according to the amount of stained biofilm observed [20].

### Pellicle formation at the air-liquid interface

Assessment of pellicle formation at the air-liquid interface was based on the CV staining assay in glass tubes as described previously [22]. Approximately 8 log_10_ CFU/ml culture were diluted 100 fold and added at 2 ml per glass tube and incubated at 25 °C for 5 days without shaking. At the end of the incubation period, supernatants were gently removed, and all tubes were washed with 3 ml per tube of PBS then allowed to dry at room temp. Tubes were then stained with 3 ml per tube of 0.1% CV for 20 min at 22 to 25 °C, washed twice with 3 ml per tube of PBS, air dried again and subsequently assessed visually for pellicle formation. Isolates were considered positive when the top surface of the culture was covered with an opaque pellicle layer attached to the wall of the tube. Quantitative measurement was performed by dissolving CV stained pellicle in 4 ml of 85% ethanol and the OD570 was measured using microplate reader at 200 μl per well.

### Tolerance of *E. coli* O26 biofilms to disinfectants

The protective effect of biofilm on isolates were assessed by exposing biofilm to quaternary ammonium compounds (QACs; Profoam, Kwiksan 22) and Peracetic acid-based sanitizer (Topactive Des.) for 2 min. All isolates that demonstrated the capacity to form biofilm on polystyrene plate after 24, 48 or 72 h at 25 °C were assessed. Isolates were allowed to form biofilm on polystyrene plate as outlined above. At the end of the incubation period, bacterial supernatants were gently aspirated and discarded, and each well was washed in triplicate with 200 μl of sterile PBS. The plates were dried and 200 μl of sterile PBS was added to three wells as an untreated control, while another three wells were filled with 200 μl of either Profoam, Kwiksan 22 or Topactive Des. and incubated for 2 min at 25 °C. At the end of the exposure time, antimicrobial agents were removed by aspiration and 170 μl of sterile Dey Engley broth (DEB; BBL, Difco, Sparks, MD) supplemented with 0.3% soytone and 0.25% sodium chloride was added to each well to neutralize the effect of disinfectants. The surface of each well was then scraped with sterile pipette tips and the contents transferred into a sterile tube. The bacterial biofilm cells were diluted and subcultured on nutrient agar for enumeration of viable cells. At least two biological replicates were performed for each isolate with PBS and disinfectants.

### Statistical analysis

Calculation of linear correlation between two variables and one way analysis of means (Tukey’s method) was performed using Minitab software (Minitab 16; Minitab Inc., Minneapolis, Minn). A “*P”* value of equal to or less than 0.01 was considered significant.

## Results

### Detection of curli and cellulose on CRI agar

**Fig. 1:**
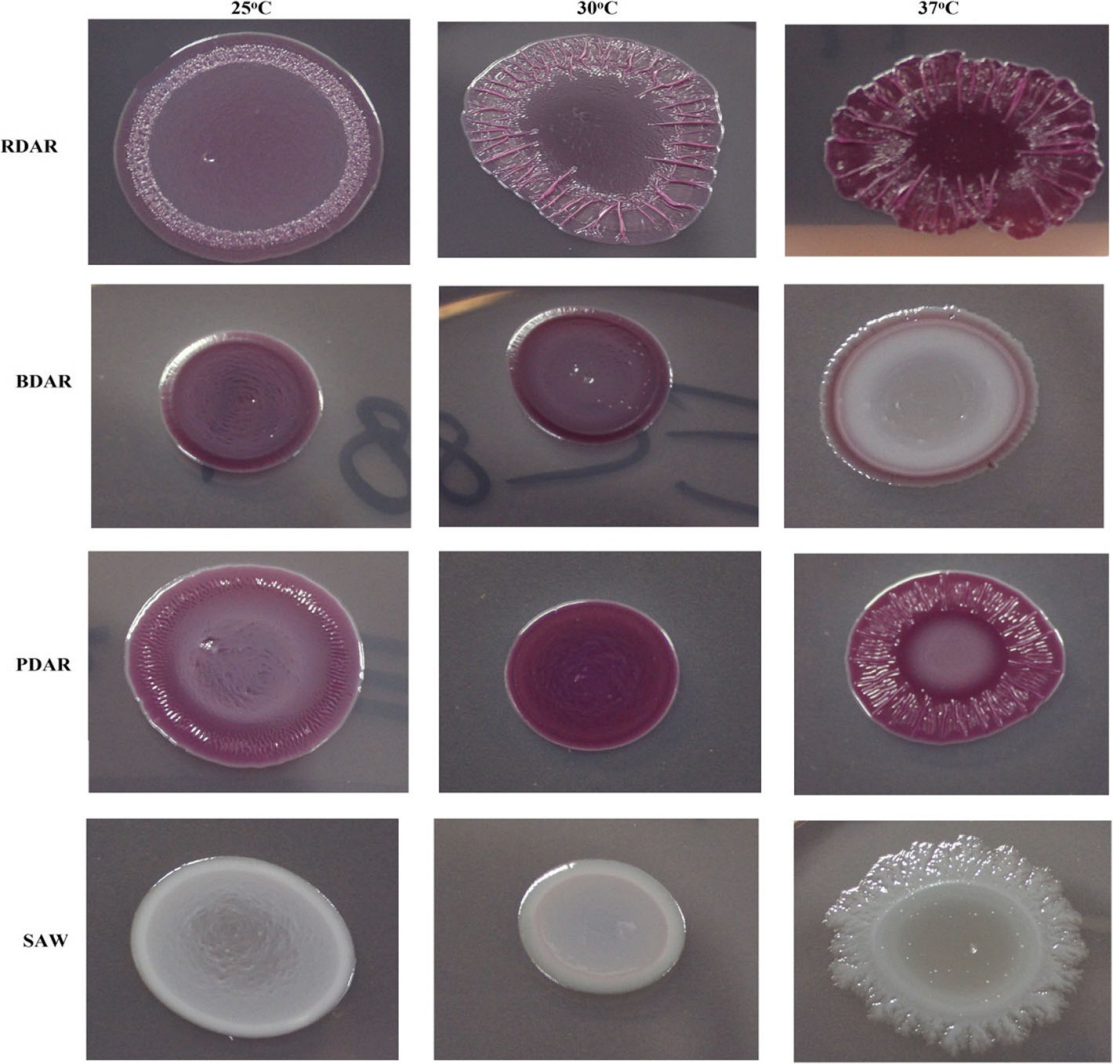
Morphotypes expressed by *E. coli* O26 isolates at 25, 30 and 37 °C. Morphotypes are: RDAR; expresses curli fimbriae and cellulose, BDAR; expresses curli fimbriae, PDAR; express cellulose, SAW; no curli fimbriae or cellulose. Cells were grown on Congo Red Indicator agar plates for 24 h at 37 °C, 48 h at 30 °C or 72 h at 25 °C

Colony morphotypes (curli, cellulose, none) were assessed on (CRI) agar plates, and representative morphotypes are shown in Fig. 1. Of the 40 *E.coli* O26 isolates assessed, 22 (55%) isolates demonstrated ability to produce at least one of the ECM components (Table 1). Regardless of the growth conditions, the ability to exhibit the BDAR morphotype (curli expression) was seldom observed with just two NTEC isolates, one pEHEC and one EHEC displaying this morphology. The PDAR morphotype was more regularly observed with 16 (59.3%) EHEC and two pEHEC isolates exhibiting this morphology. RDAR morphotype does not seem to be a common characteristic of *E. coli* O26 isolates as only a single EHEC isolate expressed both cellulose and curli and only at 37 °C. aEPEC isolates (100%) were characterized by the expression of SAW morphotypes at 25, 30 and 37 °C. In contrast, only eight EHEC isolates (29.6%) expressed SAW morphotype at tested temperatures. Comparing EHEC from human and cattle isolates, three human isolates (30%) and five cattle isolates (29.4%) showed SAW at 37 °C, 30 °C and 25 °C and two human showed BDAR at 37 °C but SAW at 30 °C and 25 °C.

**Table 1:** List of *E.coli* O26 isolates used in this study, genotypic characteristics, morphotypes, motility and *mlrA*

Strains	Source	*stx* ^*a*^	*eae*	*ehx*	*rmal-SNP*	*ecf*	Pathotype	ECM^b^	Motility	*mlrA*
								37/30/25 °C		
EC1A	Cattle	*stx1*	+	+	+	+	EHEC	SAW/SAW/SAW^c^	M^g^	Interrupted
EC1113B	Cattle	*stx1*	+	+	+	+	EHEC	PDAR/PDAR/PDAR^d^	M	Intact
EC1643B	Cattle	*stx1*	+	+	+	+	EHEC	PDAR/PDAR/PDAR	M	Intact
EC1857	Cattle	*stx1*	+	+	+	+	EHEC	PDAR/PDAR/PDAR	M	Intact
EC217	Cattle	*stx1*	+	+	+	+	EHEC	SAW/SAW/SAW	M	Intact
EC3455	Cattle	*stx1*	+	+	+	+	EHEC	RDAR^e^/BDAR/BDAR	M	Intact
EC3522	Cattle	*stx1*	+	+	+	+	EHEC	SAW/SAW/SAW	M	Intact
EC3547A	Cattle	*stx1*	+	+	+	+	EHEC	PDAR/PDAR/PDAR	M	Intact
EC3652B	Cattle	*stx1*	+	+	+	+	EHEC	BDAR^f^/PDAR/PDAR	M	Intact
EC3659B	Cattle	*stx1*	+	+	+	+	EHEC	BDAR/PDAR/PDAR	M	Intact
EC3671A	Cattle	*stx1*	+	+	+	+	EHEC	BDAR/PDAR/PDAR	M	Intact
EC3738B	Cattle	*stx1*	+	+	+	+	EHEC	SAW/SAW/SAW	M	Interrupted
EC3743A	Cattle	*stx1*	+	+	+	+	EHEC	BDAR/PDAR/PDAR	M	Intact
EC4	Cattle	*stx1*	+	+	+	+	EHEC	SAW/SAW/SAW	M	Interrupted
EC478B	Cattle	*stx1*	+	+	+	+	EHEC	BDAR/PDAR/PDAR	M	Intact
EC674	Cattle	*stx1*	+	+	+	+	EHEC	BDAR/PDAR/PDAR	M	Intact
EC7B	Cattle	*stx1*	+	+	+	+	EHEC	BDAR/PDAR/PDAR	M	Intact
EC4158QH1	Clinical	*stx1*	+	+	+	+	EHEC	SAW/SAW/SAW	M	Interrupted
EC4159QH2	Clinical	*stx1*	+	+	+	+	EHEC	SAW/SAW/SAW	M	Interrupted
EC4160QH3	Clinical	*stx1*	+	+	+	+	EHEC	BDAR/PDAR/PDAR	M	Intact
EC3213QH34	Clinical	*stx1*	+	+	+	+	EHEC	BDAR/PDAR/PDAR	M	Intact
EC4161QH4	Clinical	*stx1*	+	+	+	+	EHEC	BDAR/SAW/SAW	M	Interrupted
EC4162QH5	Clinical	*stx1*	+	+	+	+	EHEC	PDAR/PDAR/PDAR	M	Intact
EC4163QH6	Clinical	*stx1*	+	+	+	+	EHEC	BDAR/PDAR/PDAR	M	Intact
EC4164QH7	Clinical	*stx1*	+	+	+	+	EHEC	SAW/SAW/SAW	M	Intact
EC4165QH8	Clinical	*stx1*	+	+	+	+	EHEC	BDAR/SAW/SAW	M	Interrupted
EC4166QH9	Clinical	*stx1*	+	+	+	+	EHEC	PDAR/PDAR/PDAR	M	Intact
EC801	Cattle	–	+	+	+	+	pEHEC	SAW/BDAR/BDAR	M	Intact
EC3983A	Cattle	–	+	+	+	+	pEHEC	BDAR/PDAR/PDAR	M	Intact
EC3989A	Cattle	–	+	+	+	+	pEHEC	PDAR/PDAR/PDAR	M	Intact
EC3435A	Cattle	–	+	–	–	–	aEPEC	SAW/SAW/SAW	NM^h^	Intact
EC3457	Cattle	–	+	–	–	–	aEPEC	SAW/SAW/SAW	M	Intact
EC3610A	Cattle	–	+	–	–	–	aEPEC	SAW/SAW/SAW	NM	Intact
EC3727A	Cattle	–	+	–	–	–	aEPEC	SAW/SAW/SAW	NM	Intact
EC3735A	Cattle	–	+	–	–	–	aEPEC	SAW/SAW/SAW	NM	Intact
EC3768A	Cattle	–	+	–	–	–	aEPEC	SAW/SAW/SAW	NM	Intact
EC4013A	Cattle	–	+	–	–	–	aEPEC	SAW/SAW/SAW	NM	Intact
EC4039A	Cattle	–	+	–	–	–	aEPEC	SAW/SAW/SAW	M	Interrupted
EC3536B	Cattle	–	–	–	–	–	NTEC	BDAR/BDAR/BDAR	M	Intact
EC3946A	Cattle	–	–	–	–	–	NTEC	SAW/BDAR/BDAR	M	Intact

^a^All strains were negative for *stx2*

^b^ECM: extracellular matrix components

^c^SAW: no curli fimbriae or cellulose

^d^PDAR: cellulose

^e^RDAR: curli and cellulose

^f^BDAR: curli

^g^M: Motile

^h^NM: Non motile

### Motility

Evaluating *E. coli* O26 isolates for their motility on 0.3% agar revealed that 31 (77.5%) of isolates were motile. When 0.2% motility agar was used for nine isolates that did not show motility on 0.3% agar, a further three (7.5%) isolates demonstrated motility. Lack of motility was a common characteristic in aEPEC isolates with 6 (75%) isolates testing non-motile (Table 1). Motility was observed in all EHEC isolates regardless of source.

### Prophage insertion in *mlrA (yehV)*

Seven EHEC and a single aEPEC (Table 1) displayed a prophage insertion at *mlrA* (F/B = 702 bp). Isolates that carry a prophage inserted at *mlrA* was found to express the SAW morphotype at 25 °C. The percentage of EHEC from human clinical cases with a prophage insertion at *mlrA* was 40% which is higher than that detected in EHEC from cattle (17.6%).

### Cell surface hydrophobicity

**Fig. 2:**
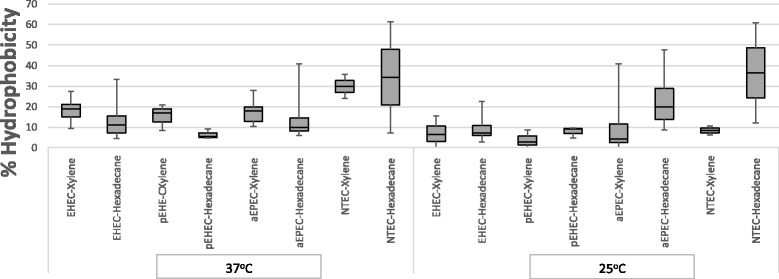
Box-plot of cell surface hydrophobicity of *E. coli* O26 pathotypes as measured at 37 and 25 °C using xylene and hexadecane. Results represent the average of percentage of bound cells to xylene and hexadecane. Data were grouped by the cell surface hydrophobicity of pathotypes. For each box, the lower hinge, upper hinge and inside line represent the 25th (Q1) percentile, the 75th (Q3) percentile and the median, respectively

The mean hydrophobicity values of *E. coli* O26 isolates of EHEC, pEHEC, aEPEC and NTEC at 37 and 25 °C are shown in Fig. 2. The percentage of bound cells to xylene and hexadecane was determined at 25 and 37 °C by BATH assay. Overall, the mean hydrophobicity (%) values of isolates obtained with xylene were shown to be higher at 37 °C (18.7%) than at 25 °C (1.7%). In contrast, no significant differences was observed in the mean hydrophobicity values of isolates using hexadecane at 25 °C (13.2%) or 37 °C (12.7%). Among the four pathotypes, NTEC had significantly higher mean hydrophobicity values than aEPEC, EHEC and pEHEC at 37 °C. When hydrophobicity was determined at 25 °C, NTEC and aEPEC were significantly more hydrophobic than EHEC and pEHEC. Hydrophobicity measurements obtained for human and cattle EHEC strains showed no significant differences at 37 or 25 °C regardless of the hydrocarbon used to determine their cell surface hydrophobicity.

### Biofilm formation on polystyrene microtiter plates

**Fig. 3:**
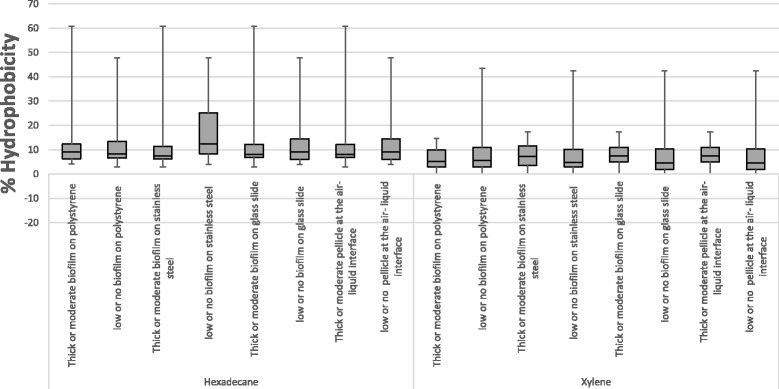
Box-plot of cell surface hydrophobicity of *E. coli* O26 isolates as measured at 25 °C using hexadecane and xylene and its influence on biofilm formation. Results represent the average of percentage of bound cells to hexadecane and xylene. Data were grouped by the capacity of biofilm formation on polystyrene microtiter plates, stainless steel coupons and glass slides and pellicle formation at the air-liquid interface. For each box, the lower hinge, upper hinge and inside line represent the 25th (Q1) percentile, the 75th (Q3) percentile and the median, respectively. Lower and upper bars represent the lower and the upper whiskers respectively

Assessment of biofilm formation on polystyrene microtiter plates showed that the ability to form biofilm and the quantity of biofilm produced for the forty *E. coli* O26 isolates vary after incubation for 24, 48, or 72 h. After incubation for 24 h, only four (three EHEC and a single aEPEC) isolates showed biofilm production and all four were classified as low producers (OD_570_ > 0.08- ≤ 0.16). After incubation for 48 h, 20 isolates displayed biofilm formation with 14 isolates classified as low producers (OD_570_ > 0.078- ≤ 0.156), four isolates were moderate producers (OD_570_ > 0.156- ≤ 0.312) and thick biofilm formation was observed with two isolates (OD_570_ > 0.312). After incubation for 72 h, 28 isolates displayed capability to produce biofilm on polystyrene plates of which 15 were categorised as low producers (OD_570_ > 0.071- ≤ 0.142), six isolates were moderate biofilm producers (OD_570_ > 0.142- ≤ 0.284) and seven isolates developed thick biofilm mass(OD_570_ > 0.284). EHEC were significantly more likely to produce biofilms after incubation for 48 h in comparison to aEPEC strains, however these differences did not persist at 72 h. Nonetheless, thick biofilm mass was observed in 33.3% of EHEC in comparison to 12.5% of aEPEC. Considering human and cattle isolates, eight (80%) EHEC human clinical isolates and 14 (82.3%) EHEC cattle isolates were able to form biofilm. When comparing the importance of attributes namely ECM components, motility, intact *mlrA* gene and hydrophobicity in biofilm formation, it was observed that these attributes were significantly more likely to be expressed by strong and moderate biofilm formers and 48 h biofilm producers than lower or biofilm-deficient isolates, (*P* value < 0.001). In addition, the low to limited capacity of biofilm formation at 25 °C was associated with insertion in *mlrA* or lack of ECM morphotype in EHEC but with lack of motility, expression of SAW morphotype and interrupted *mlrA* in aEPEC (Table 1). It was also observed that there was little overlap between impairments in these attributes. For example, SAW morphotype in EHEC non-biofilm forming isolates were observed along with interrupted *mlrA* in five isolates and SAW morphotype were displayed along with lack of motility in five aEPEC isolates. An exception of this was EC4164QH7 which had mutation in RpoS (data not published) and EC4165QH8 which had both mutation in RpoS (data not published) and interrupted *mlrA* but were able to form moderate biofilm mass after 48 h. Finally, cell surface hydrophobicity had no observed impact on biofilm formation (Fig. 3).

### Biofilm formation on stainless steel coupons and glass slides

The results of biofilm formation on stainless steel coupons and glass slides by *E. coli* O26 isolates in this study are shown in Table 2 (Additional file 1: Table S1). The majority of the isolates had the capacity to form biofilms on the surface of stainless steel coupons (87.5%) and glass slides (95%) at the air-liquid interface. In terms of pathotypes, 27 EHEC (100%), three pEHEC (100%) and two NTEC (100%) were significantly more likely to produce biofilms on stainless steel at the air-liquid interface after 72 h in comparison to three aEPEC isolates (37.5%). However, these differences did not persist when glass slides were used to investigate biofilm formation with six aEPEC (75%) forming a biofilm on glass slides. Comparative analysis of biofilm formation by human and cattle EHEC did not identify differences in biofilm formation between sources. Isolates with these profiles: motile, ECM components (curli and or cellulose), and intact *mlrA* showed thick biofilm mass at the air-liquid interface (score 2 or 3) on stainless steel and glass slides than isolates that lacked these profiles, (*P* value< 0.001). The possible influence of hydrophobicity on biofilm formation was also investigated; however, no correlation was found (Fig. 3).

**Table 2:** Biofilm formation on polystyrene microtiter plates, stainless steel coupons, glass slides and pellicle formation at the air-liquid interface

Pathotypes	Isolates No.	Biofilm mass on polystyrene at incubation time of			Biofilm mass on^b^		Pellicle formation^c^
		24 h	48 h	72 h	SS	GS	
EHEC	EC1A	0.024 ± 0.004^a^	0.076 ± 0.015	0.049 ± 0.007	1	1	0.061 ± 0.011
	EC1113B	0.029 ± 0.005	0.119 ± 0.022	0.191 ± 0.039	3	3	1.884 ± 0.259
	EC1643B	0.047 ± 0.006	0.251 ± 0.033	1.126 ± 0.153	3	3	3.595 ± 0.191
	EC1857	0.036 ± 0.006	0.063 ± 0.011	0.107 ± 0.016	3	3	0.856 ± 0.078
	EC217	0.054 ± 0.009	0.100 ± 0.015	0.043 ± 0.008	2	1	0.025 ± 0.009
	EC3455	0.066 ± 0.008	0.025 ± 0.005	0.060 ± 0.007	3	3	1.228 ± 0.145
	EC3522	0.060 ± 0.008	0.058 ± 0.010	0.086 ± 0.011	1	1	0.039 ± 0.034
	EC3547A	0.043 ± 0.003	0.236 ± 0.014^c^	0.503 ± 0.030	2	2	0.839 ± 0.113
	EC3652B	0.056 ± 0.010	0.102 ± 0.014	0.104 ± 0.016	3	3	1.875 ± 0.103
	EC3659B	0.026 ± 0.009	0.058 ± 0.009	0.093 ± 0.014	3	3	1.652 ± 0.152
	EC3671A	0.016 ± 0.005	0.091 ± 0.010	0.125 ± 0.017	3	3	1.554 ± 0.167
	EC3738B	−0.010 ± 0.003	0.032 ± 0.013	0.093 ± 0.027	1	1	0.145 ± 0.030
	EC3743A	0.038 ± 0.006	0.087 ± 0.011	0.075 ± 0.010	3	3	1.218 ± 0.087
	EC4	0.023 ± 0.004	0.045 ± 0.009	0.024 ± 0.005	2	1	0.097 ± 0.023
	EC478B	0.086 ± 0.011^b^	0.127 ± 0.011	0.133 ± 0.011	2	2	0.480 ± 0.061
	EC674	0.011 ± 0.005	0.064 ± 0.013	0.130 ± 0.023	3	3	1.660 ± 0.151
	EC7B	0.082 ± 0.014	0.109 ± 0.013	0.230 ± 0.052	3	3	1.397 ± 0.091
	EC4158QH1	0.047 ± 0.005	0.082 ± 0.015	0.069 ± 0.011	1	1	0.092 ± 0.042
	EC4159QH2	0.049 ± 0.007	0.089 ± 0.013	0.055 ± 0.007	2	1	0.035 ± 0.025
	EC4160QH3	0.098 ± 0.015	0.034 ± 0.008	0.044 ± 0.008	2	3	2.257 ± 0.099
	EC3213QH34	0.036 ± 0.006	0.072 ± 0.007	0.171 ± 0.017	3	3	1.079 ± 0.046
	EC4161QH4	0.075 ± 0.012	0.060 ± 0.010	0.061 ± 0.009	2	1	0.050 ± 0.021
	EC4162QH5	0.001 ± 0.009	0.088 ± 0.020	1.021 ± 0.154	3	3	2.882 ± 0.255
	EC4163QH6	0.017 ± 0.003	0.135 ± 0.030	1.469 ± 0.304	2	3	1.848 ± 0.127
	EC4164QH7	0.053 ± 0.006	0.223 ± 0.025	0.245 ± 0.025	2	1	0.092 ± 0.034
	EC4165QH8	0.070 ± 0.008	0.127 ± 0.019	0.156 ± 0.026	1	1	0.065 ± 0.037
	EC4166QH9	0.061 ± 0.007	0.051 ± 0.010	0.070 ± 0.010	3	3	2.746 ± 0.163
pEHEC	EC801	0.040 ± 0.005	0.119 ± 0.017	0.121 ± 0.020	2	1	0.150 ± 0.042
	EC3983A	0.033 ± 0.006	0.057 ± 0.011	0.074 ± 0.010	2	1	0.081 ± 0.024
	EC3989A	0.063 ± 0.008	0.638 ± 0.036^d^	1.402 ± 0.203	3	3	3.859 ± 0.138
aEPEC	EC3435A	0.044 ± 0.009	0.041 ± 0.007	0.060 ± 0.007	0	1	0.104 ± 0.064
	EC3457	0.110 ± 0.020	0.134 ± 0.023	0.135 ± 0.014	1	1	−0.001 ± 0.030
	EC3610A	0.019 ± 0.003	0.017 ± 0.004	0.033 ± 0.005	1	0	0.018 ± 0.023
	EC3727A	0.037 ± 0.005	0.034 ± 0.007	0.119 ± 0.021	0	1	0.268 ± 0.051
	EC3735A	0.046 ± 0.011	0.017 ± 0.011	0.108 ± 0.019	0	1	0.135 ± 0.027
	EC3768A	0.035 ± 0.012	0.078 ± 0.017	0.206 ± 0.028	0	1	0.128 ± 0.020
	EC4013A	0.048 ± 0.018	0.027 ± 0.011	0.101 ± 0.014	0	1	0.177 ± 0.032
	EC4039A	0.006 ± 0.003	0.060 ± 0.009	0.048 ± 0.007	1	0	−0.005 ± 0.013
NTEC	EC3536B	0.052 ± 0.005	0.224 ± 0.021	0.475 ± 0.034	3	3	0.966 ± 0.158
	EC3946A	0.052 ± 0.006	0.313 ± 0.056	0.404 ± 0.034	3	3	0.587 ± 0.127
Negative control	Lb broth	0.080 ± 0.0009	0.078 ± 0.001	0.071 ± 0.0004	0	0	

^a^Values are shown as mean of biofilm production ± standard error on polystyrene surfaces, SS: stainless steel, GS: glass slide at 25 °C. According to the biofilm mass quantified with crystal violet staining assay at 570 nm isolates were labelled as the following: low, medium and thick biofilm formers

^b^Visible biofilms on stainless steel and glass slides and was scored as 0: no visible biofilm, scored on a scale from 1 to 3 to a thick biofilm at the air-liquid

^c^The presence and absence of visible pellicles biofilms was scored visually before staining with CV

### Pellicle formation at the air-liquid interface

The presence of visible pellicles biofilms at the air-liquid interface was scored visually before staining with CV. When isolates were tested for their capacity to form a pellicle layer attached to the wall of a glass tube at the air-liquid interface, 20 of 40 isolates (50%) displayed pellicle formation at the air-liquid interface. At the pathotype level, 17 EHEC ranked moderate to high pellicle producers in comparison to aEPEC which did not form a biofilm layer at the air-liquid interface. A single pEHEC isolate and both NTEC isolates formed thick pellicles. In addition, a significant correlation was observed between thick biofilm producers on polystyrene, stainless steel and glass slides and pellicle formation. However, pellicle formation was not an indicator for biofilm formation on those surfaces. Examining factors associated with biofilm formation revealed that motile isolates expressing cellulose or curli and harbouring intact *mlrA* were capable of producing well attached pellicle at the air-liquid interface as opposed to strains that did not exhibit these characteristics, (*P* value < 0.001). Finally, no correlation was found between cell surface hydrophobicity and pellicle formation (Fig. 3).

### Tolerance of EHEC O26 biofilm cells to antimicrobial agents

**Fig. 4:**
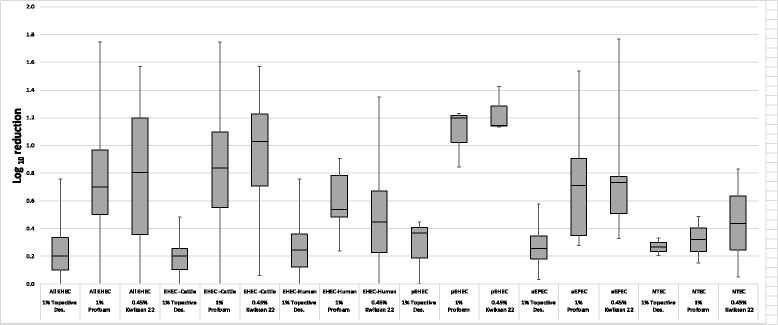
Box-plot of log_10_ reduction of *E. coli* O26 after 2 min exposure to Kwiksan 22, Profoam and Topactive Des. based on isolates source and pathotypes. For each box, the lower hinge, upper hinge and inside line represent the 25th (Q1) percentile, the 75th (Q3) percentile and the median, respectively. Lower and upper bars represent the lower and the upper whiskers respectively

The influence of disinfectants on *E. coli* O26 cell viability within the biofilm was determined by enumerating viable cells remaining after 0.45% Kwiksan 22 (QAC), 1% Profoam (QAC) and 1% Topactive Des. treatment (Table 3, Additional file 2: Table S2). After 2 min exposure, none of the disinfectants were able to completely inactivate all cells within a biofilm. Exposure to 1% Topactive Des. resulted in 0.03 to 0.76 log_10_ reduction. Treatment with 1% Profoam had a greater effect on biofilm cells, led to 0.02–1.74 log_10_ reductions. When isolates were exposed for 2 min to 0.45% Kwiksan 22 (QAC) biofilm cells reduction ranged from 0.05–1.77 log_10_ CFU per well. In terms of pathotypes, Kwiksan 22 has shown to be the most effective sanitizer against all pathotypes whereas Topactive Des. was the least effective (Fig. 4). Among pathotypes, the mean reduction caused by Kwiksan was greatest in pEHEC (1.145 log_10_ CFU/well) and lowest in those of NTEC (0.44 log_10_ CFU/well). Profoam resulted in the same mean log_10_ CFU/well reduction for both EHEC (0.70 log_10_ CFU/well) and aEPEC (0.70 log_10_ CFU/well) but had a greater mean reduction level in pEHEC (1.19 log_10_ CFU/well) and NTEC (0.32 log_10_ CFU/well). In contrast, when biofilm formed by all pathotypes were treated with Topactive Des. the mean of viable cell counts was reduced by 0.2 to 0.3 log_10_ CFU/well. Statistical analysis of means indicated that pathotypes, biofilm density, production of one or both of the extracellular components had no impact on *E. coli* O26 biofilm cells survival to disinfectants treatment. Human clinical and cattle isolates showed various level of tolerance to disinfectant with cattle isolates were being more susceptible to disinfectant intervention than their human counterparts (Fig. 4) although not statistically significant.

**Table 3:** Exposure of *E. coli* O26 biofilms to Topactive Des., Kwiksan 22 and Profoam disinfectants for 2 min

Pathotypes	Isolates No.	2 min exposure to sanitization treatment					
		PBS	Topactive DES	PBS	Kwiksan	PBS	Profoam
EHEC	EC1113B	7.094 (±0.077)	6.944 (±0.166)	6.77 (±0.16)	6.71 (±0.02)	7.31±0.05)	5.56 (±0.78)
	EC1643B	6.644 (±0.515)	6.452 (±0.442)	7.04 (±0.14)	5.47 (±0.62)	6.99 (±0.34)	6.11 (±0.19)
	EC1857	7.005 (±0.194)	6.745 (±0.297)	6.60 (±0.39)	6.25 (±0.49)	6.58 (±0.43)	6.58 (±0.47)
	EC217	6.994 (±0.166)	6.906 (±0.176)	6.58 (±0.22)	5.34 (±0.53)	6.29 (±0.34)	5.77 (±0.50)
	EC3213QH34	6.609 (±0.132)	6.430 (±0.457)	6.82 (±0.31)	6.75 (±0.18)	6.71 (±0.53)	6.22 (±0.52)
	EC3522	6.081 (±0.249)	6.055 (±0.464)	6.22 (±0.36)	5.46 (±0.46)	6.18 (±0.26)	5.53 (±0.27)
	EC3547A	6.476 (±0.248)	6.425 (±0.285)	6.58 (±0.34)	5.50 (±0.80)	6.29 (±0.55)	6.27 (±0.51)
	EC3652B	6.601 (±0.383)	6.390 (±0.370)	6.55 (±0.53)	5.36 (±0.74)	6.30 (±0.41)	5.67 (±0.41)
	EC3659B	6.649 (±0.485)	6.403 (±0.584)	6.25 (±0.25)	5.04 (±0.11)	6.21 (±0.90)	4.53 (±1.05)
	EC3671A	6.648 (±0.171)	6.170 (±0.086)	7.11 (±0.30)	6.26 (±1.01)	6.84 (±0.10)	5.85 (±0.75)
	EC3738B	7.346 (±0.075)	6.971 (±0.120)	6.79 (±0.19)	5.27 (±0.21)	6.37 (±0.07)	5.25 (±0.33)
	EC3743A	7.250 (±0.250)	7.250 (±0.250)	6.53 (±0.43)	5.84 (±0.79)	6.72 (±0.71)	5.93 (±1.15)
	EC478B	6.684 (±0.430)	6.521 (±0.354)	7.15 (±0.05)	5.70 (±0.63)	6.67 (±0.36)	5.14 (±0.86)
	EC674	6.455 (±0.783)	6.241 (±0.879)	6.45 (±0.55)	6.14 (±0.88)	6.70 (±0.54)	6.66 (±0.52)
	EC7B	6.951 (±0.326)	6.468 (±0.210)	6.48 (±0.61	5.50 (±0.01)	6.77 (±0.18)	5.71 (±0.33)
	EC4158QH1	7.104 (±0.125)	7.019 (±0.045)	6.45 (±0.31)	5.92(±0.43)	6.51 (±0.37)	5.96 (±0.64)
	EC4159QH2	6.679 (±0.187)	6.333 (±0.294)	6.94 (±0.18)	5.59 (±0.51)	6.71 (±0.10)	6.19 (±0.02)
	EC4160QH3	6.207 (±0.298)	5.812 (±0.665)	6.00 (±0.35)	6.00 (±0.35)	5.96 (±0.12)	5.72 (±0.48)
	EC4162QH5	6.756 (±0.078)	6.620 (±0.202)	6.12 (±0.65)	5.58 (±0.94)	6.76 (±0.25)	6.30 (±0.27)
	EC4163QH6	6.878 (±0.410)	6.569 (±0.420)	6.42 (±0.47)	6.14 (±0.51)	6.65 (±0.51)	5.90 (±0.67)
	EC4164QH7	5.006 (±0.474)	4.247 (±0.145)	5.56 (±0.93)	5.19 (±0.83)	6.21 (±0.38)	5.31 (±0.09)
	EC4165QH8	6.000 (±1.000)	6.000 (±1.000)	5.89 (±0.60)	4.82 (±1.10)	6.93 (±0.20)	6.05 (±0.69)
pEHEC	EC801	6.397 (±0.320)	5.950 (±0.777)	6.19 (±0.27)	5.04 (±0.68)	6.31 (±0.46)	5.11 (±0.99)
	EC3983A	6.740 (±0.550)	6.370 (±0.630)	6.38 (±0.16)	4.95 (±0.59)	6.44 (±1.01)	5.60 (±0.98)
	EC3989A	7.161 (±0.140)	7.157 (±0.074)	7.35 (±0.10)	6.21 (±0.11)	7.36 (±0.07)	6.13 (±0.05)
aEPEC	EC3457	6.253 (±0.332)	5.679 (±0.390)	6.48 (±0.46)	4.71 (±1.25)	6.47 (±0.50)	4.93 (±1.38)
	EC3727A	6.302 (±0.085)	6.270 (±0.044)	6.18 (±0.41)	5.45 (±0.32)	6.42 (±0.26)	5.52 (±0.04)
	EC3735A	6.466 (±0.256)	6.285 (±0.386)	6.76 (±0.28)	5.98 (±0.32)	6.42 (±0.22)	5.72 (±0.40)
	EC3768A	6.097 (±0.198)	5.842 (±0.050)	6.20 (±0.95)	5.87 (±1.32)	6.31 (±0.40)	6.03 (±0.57)
	EC4013A	6.491 (±0.300)	6.145 (±0.173)	5.99 (±0.18)	5.49 (±0.73)	6.24 (±0.69)	5.89 (±0.89)
NTEC	EC3536B	6.812 (±0.567)	6.609 (±0.443)	6.48 (±0.60)	5.65 (±1.00)	6.52 (±0.39)	6.37 (±0.28)
	EC3946A	7.339 (±0.138)	7.006 (±0.155)	7.05 (±0.05)	7.00 (±0.00)	7.23 (±0.02)	6.75 (±0.07)

Biofilms were formed in polystyrene plates and data are shown as mean log_10_ CFU per well (±the standard errors of the means). The influence of disinfectants on biofilms was determined by enumerating viable cells remaining after treatment with 1% Topactive Des., 0.45% Kwiksan 22 (QAC) and 1% Profoam (QAC) treatment and compared to that of PBS control

## Discussion

EHEC of serogroup O26 have been associated with foodborne disease outbreaks worldwide [7, 8, 37]. Formation and encasement of *E. coli* O26 cells in a complex biofilm matrix may enhance resistance to antimicrobials agents under various conditions (reviewed in Srey et al. [38]). There are a number of described methods for biofilm assessment on abiotic surfaces. However, no standard accepted biofilm methodology has been published to date. In this study we choose to utilize the low salt (5 g/L) Luria-Bertani broth (LS-LB) for a number of reasons. An increase in the number of adherent cells was seen with *E. coli* strains in nutrient-rich medium such as LS-LB, without salt LB or tryptic soy broth (TSB) while low capacity of biofilm formation was observed in nutrient-defined medium such as (M9) and diluted meat juice (DMJ) [32, 39]. In addition, Bokranz et al. [32] observed that biofilm formation in LB medium without salt correlated with the colony morphotype on CRI agar plates. The use of LB media and protocols previously utilized to study biofilm phenotypes in *E. coli* will facilitate comparison with other studies when possible.

The current study investigated whether biofilm production is associated with particular pathotypes, curli and/or cellulose production, motility, intact *mlrA,* and hydrophobicity. Results presented here demonstrated that a prophage insertion in *mlrA*, lack of motility and failure in producing ECM prevented or lowered biofilm formation with overlapping between these attributes was observed. In EHEC, curli expression was observed more frequently at 37 °C but cellulose expression was the most predominant morphotype at 30 and 25 °C. In addition, isolates produced cellulose or curli at 25 °C were able to produce biofilm on at least one of the tested surfaces (Table 2, Additional file 1: Table S1). This is in agreement with a previous finding of Uhlich et al. [40] who found that in EHEC non-O157, cellulose is suppressed at 37 °C but produced at 30 °C and 25 °C and that production of cellulose or curli or both were associated with biofilm production. In aEPEC isolates, SAW morphotype was the predominant characteristic at all tested temperatures and low biofilm formation was observed, in contrast; BDAR was displayed almost at all temperatures in NTEC (Table 1) and strong biofilm formation was observed. The obtained result could be due to the fact that both curli (BDAR) and cellulose (PDAR) production are dependent on *csgD* which is influenced by temperature (the only variable tested here), pH and available nutrients. In addition, the insertion of a prophage in *mlrA* in eight isolates (Table 1) and mutation in RpoS in only two isolates (EC4164QH7 and EC 4165QH8; data not published) could explain the production of SAW morphotype by a number of isolates. Finally, lack of motility was observed only in aEPEC (75%) and could be another attribute that limit ECM expression and biofilm formation. It has been reported that lack of motility could inhibit biofilm formation by preventing curli expression in bacteria that could not be complemented for curli by restoring *mlrA* [25]. However, the remaining isolates (Table 1) that were motile with intact *mlrA* and wild type RpoS (all isolates had wild type RpoS except EC4164QH7 and EC4165QH8; data not published) but exhibited SAW morphotype could have additional, yet to be discovered structural or regulatory gene mutations.

The influence of physiochemical properties represented by cell surface hydrophobicity on biofilm formation using BATH assay was also investigated. The bacterial adhesion to xylene and hexadecane has been extensively used for measuring cell surface hydrophobicity [20, 24, 32, 34]. Comparing cell surface hydrophobicity by measuring adherence to hydrocarbons showed differences in the affinity to the two chemicals. Differences in the degree of adherence to xylene and hexadecane have been previously observed [34]. It was speculated that each hydrocarbon might measure different aspects of hydrophobicity [24, 34]. Among the four pathotypes tested in this study, NTEC isolates had significantly higher hydrophobicity values than other pathotypes at all tested temperatures. The high hydrophobicity values for *eae*-negative isolates is in agreement with others [20, 34] who reported the same observation for *eae*-negative isolates of O103:H2 serotype and O157:HR [34]. In addition, no correlation was found between cell surface hydrophobicity and biofilm formation on any of the surfaces tested or at the air-liquid interface (Fig. 3), which is consistent with previous studies [34, 41, 42]. Together with the results of this study, this suggests that the adhesion process is likely to involve a variety of physiochemical and/or biological factors [24, 43].

The capacity of biofilm formation at 25 °C in *E. coli* O26 isolates which were *stx*^*+*^ (EHEC) and *stx*^−^ (pEHEC, NTEC and aEPEC), with various ECM, *mlrA* and motility profiles on polystyrene plates after incubation for 24, 48 and 72 h and on stainless steel and glass slide was also investigated in this study. The results are in accordance with previous studies of Uhlich et al. (2013) and Chen et al. (2013) [22, 23] who observed a positive role for curli and/or cellulose, motility and intact *mlrA* in biofilm formation. Biofilm development for motile isolates with intact *mlrA* and expressing cellulose or curli occurred earlier than other isolates regardless of pathotypes on polystyrene surface and developed thick biofilm mass on stainless steel or glass slides, suggesting that the presence of these components is an advantageous characteristic for biofilm formation. However, further studies using knockout mutants are required to confirm the role of the abovementioned factors on biofilm formation.

Association between ECM production, motility, intact *mlrA* and pellicle formation at the air-liquid interface was observed in this study. This is in agreement with the observation of Wang et al. (2012) who has shown that the curli-positive strains of serotype O26:H11 exhibited an overall high potency of pellicle formation [22]. In addition, isolates that exhibited the SAW phenotype were limited in their capacity to form pellicle at the air-liquid interface in this study. These findings correlate with previous reports [23] and suggest that ECM expression and biofilm formation by strains with SAW morphotypes are more inducible upon exposure to solid surfaces such as glass and stainless steel than at the air liquid interface under the conditions used in this study [23].

Moreover, biofilm production on one surface may not correlate with biofilm formation on a different surface. For example, biofilm formation of *E. coli* O26 isolates on glass slides at the air- liquid interface (95%) was significantly higher than that on polystyrene plates (70%) and pellicle formation at the air-liquid interface (50%). This finding is in agreement with previous studies where some strains of non-O157 that formed biofilm on one surface were not able to develop biofilm on other surfaces [20, 24, 44] and suggests that cell contact surfaces can influence biofilm formation. As the abiotic surfaces are commonly used in the food industry it may be necessary to evaluate specific surfaces for their capacity to act as a matrix for biofilm formation. Furthermore, biofilm formation on various surfaces seems to be a common characteristic not only for EHEC, but also for pEHEC (positive for all EHEC markers except stx) and NTEC pathotypes. It is well documented that *stx* negative pathotypes can become EHEC via acquisition of *stx* [45–47]. In addition, biofilms were demonstrated to act as an environment for dissemination of *stx* and emergence of new pathogenic strains [20]. Together with the results of this study, biofilm formation by *stx* negative isolates warrants additional investigation to determine the clinical importance of biofilm formation by this group. Finally, both human clinical and cattle isolates of EHEC pathotype were able to form biofilm which may suggest that cattle isolates represent a source of biofilm-forming bacteria that might occupy food contact surfaces, although additional factors that represent the food and meat processing environments should be considered [39, 48–51].

EHEC O26 can cause illness range from diarrhoea to severe sequelae such as HUS; therefore interventions to control this pathogen and prevent future outbreaks of illness are required. When *E. coli* O26 isolates were challenged to determine the impact of biofilm formation on sensitivity toward the tested disinfectants, a protective effect of biofilm was observed. Interestingly, strains that showed lowest biofilm formation on polystyrene plate were equally resistant to disinfectant intervention as strains that formed a dense biofilm mass (Table 3, Additional file 2: Table S2). This is consistent with the study of Vogeleer [21] where the amount of biofilm mass and expression of cellulose or curli had no impact on the ability of biofilm cells to survive disinfectants treatments. In contrast to other studies [22, 23, 52] that suggested curli and/or cellulose appeared to play a critical role in EHEC tolerance to disinfectants. Variations between studies could be attributed to the differences in experimental designs and/or the use of different bacterial strains. Additionally, differences in the response to disinfectants was observed between isolates within the same pathotypes in this study, with previous studies also reporting variation in tolerances amongst *E. coli* serogroups including *E. coli* O26 [21–23]. In our previous study [31], we showed that *E. coli* O26 planktonic cells from human and cattle could not survive the challenge with QAC and peracetic acid based disinfectants approved for use in Australian food industry at their recommended concentration, regardless of pathotypes. In the current study, although biofilms provided a protective effect to *E. coli* O26 strains against the three sanitizers, previously shown to successfully control the growth of their planktonic counterparts, the majority of isolates did not form biofilm after 24 h of incubation. Taking these findings together suggests that regular and proper sanitization should be effective to prevent the formation of biofilms in food production environments. However, it is also indicated that other factors such as the pre-conditioning of the substratum, to which the bacteria would attach could increase or inhabit the attachment [39, 48–51]. For example, pre-exposure of the food surfaces to beef juice extract provides a protective matrix for the bacterial cells impeded in [53]. In addition, the co-existence with other resistant species in a biofilm would mean that expression of resistance by a species within mixed-biofilm community could provide resistance to the whole community [48]. Furthermore, integration into a biofilm matrix could enhance the opportunities for pathogens that are non-biofilm formers and metabolically inactive cells to survive in food and meat processing environments [49, 54, 55].

## Conclusion

The study provided insight into the biofilm characteristics of EHEC that caused human infections and those from cattle origin, and other pathotypes. Some factors that appear to enhance or limit biofilm formation in *stx* positive or *stx* negative *E. coli* O26 pathotypes have been also demonstrated. Pathotypes of *E. coli* O26 showed varying capacities to form biofilms, however, most EHEC strains had the capacity to form biofilm on all surfaces and at the air-liquid interface under the conditions used in this study. The ability of biofilm formation provided a protective effect to *E. coli* O26 strains against the three sanitisers, previously shown to successfully control the growth of their planktonic counterparts. While there are caveats to the results observed in this study, the utility of this study is to provide initial insights into factors that could possibly influence biofilm formation by *E. coli* O26 and then the effect of this phenotype on tolerance to disinfectants. Further studies that represent the food and meat processing environments by considering the effect of co-existence with other microorganisms, presence of organic residues on food surfaces and resistance or adaptation to disinfection are required.

## Additional files

Additional file 1: **Table S1.** Table 2, Biofilm formation on polystyrene microtiter plates, stainless steel coupons, glass slides and pellicle formation at the air-liquid interface. (XLSX 61 kb)

Additional file 2 **Table S2.** Table 3, Exposure of *E. coli* O26 biofilms to Topactive Des., Kwiksan 22 and Profoam disinfectants for 2 min. (XLSX 22 kb)

## Abbreviations

aEPEC: Enteropathogenic *E. coli*
ATCC: American Type Culture Collection
*bfp*: bundle forming pilus
*csgD*: DNA binding transcription factor
*eae*: *E. coli* attachment and effacing gene
*ecf*: *eae* positive conserved fragments
ECM: Extra cellular matrix
EHEC: Enterohaemorrhagic *Escherichia coli*
*ehx*: Enterohaemolysin
HC: Haemolytic colitis
HUS: Haemolytic uremic-syndrome
*mlrA*: MerR-like regulator
NTEC: Non-toxigenic *E. coli*
PCR: Polymerase chain reaction
pEHEC: Potential EHEC
PFGE: Pulse-field gel electrophoresis
QACs: Quaternary Ammonium Compound
RFLP: Restriction fragment length polymorphism
RpoD: RNA polymerase sigma factor D
RpoS: RNA polymerase sigma factor S
SNP: Single nucleotide polymorphism
USA: United State of America
