# Correction to: Characterization of biofilm-forming capacity and resistance to sanitizers of a range of E.coli O26 pathotypes from clinical cases and cattle in Australia

**DOI:** 10.1186/s12866-018-1202-z

**Published:** 2018-06-19

**Authors:** Salma A. Lajhar, Jeremy Brownlie, Robert Barlow

**Affiliations:** 1 School of Environment and Science, Griffith University, Brisbane, QLD Australia; 2 Present address: CSIRO Agriculture and Food, 39 Kessels Rd, Coopers Plains, Brisbane, QLD 4108 Australia

## Correction

1. On page 4 of the original publication [1], the correct sentence should read:
  Quantitative measurement was performed by dissolving CV stained pellicle in 4 ml of 85% ethanol and the OD_570_ was measured using microplate reader at 200 μl per well.
2. Table 2 of this original publication contained some errors. The updated Table 2 is published in this correction article.

**Table 2:** Biofilm formation on polystyrene microtiter plates, stainless steel coupons, glass slides and pellicle formation at the air-liquid interface

Pathotypes	Isolates No.	Biofilm mass on polystyrene at incubation time of			Biofilm mass on^b^		Pellicle formation^c^
		24 h	48 h	72 h	SS	GS	
EHEC	EC1A	0.024 ± 0.004^a^	0.076 ± 0.015	0.049 ± 0.007	1	1	0.061 ± 0.011
	EC1113B	0.029 ± 0.005	0.119 ± 0.022	0.191 ± 0.039	3	3	1.884 ± 0.259
	EC1643B	0.047 ± 0.006	0.251 ± 0.033	1.126 ± 0.153	3	3	3.595 ± 0.191
	EC1857	0.036 ± 0.006	0.063 ± 0.011	0.107 ± 0.016	3	3	0.856 ± 0.078
	EC217	0.054 ± 0.009	0.100 ± 0.015	0.043 ± 0.008	2	1	0.025 ± 0.009
	EC3455	0.066 ± 0.008	0.025 ± 0.005	0.060 ± 0.007	3	3	1.228 ± 0.145
	EC3522	0.060 ± 0.008	0.058 ± 0.010	0.086 ± 0.011	1	1	0.039 ± 0.034
	EC3547A	0.043 ± 0.003	0.236 ± 0.014	0.503 ± 0.030	2	2	0.839 ± 0.113
	EC3652B	0.056 ± 0.010	0.102 ± 0.014	0.104 ± 0.016	3	3	1.875 ± 0.103
	EC3659B	0.026 ± 0.009	0.058 ± 0.009	0.093 ± 0.014	3	3	1.652 ± 0.152
	EC3671A	0.016 ± 0.005	0.091 ± 0.010	0.125 ± 0.017	3	3	1.554 ± 0.167
	EC3738B	−0.010 ± 0.003	0.032 ± 0.013	0.093 ± 0.027	1	1	0.145 ± 0.030
	EC3743A	0.038 ± 0.006	0.087 ± 0.011	0.075 ± 0.010	3	3	1.218 ± 0.087
	EC4	0.023 ± 0.004	0.045 ± 0.009	0.024 ± 0.005	2	1	0.097 ± 0.023
	EC478B	0.086 ± 0.011	0.127 ± 0.011	0.133 ± 0.011	2	2	0.480 ± 0.061
	EC674	0.011 ± 0.005	0.064 ± 0.013	0.130 ± 0.023	3	3	1.660 ± 0.151
	EC7B	0.082 ± 0.014	0.109 ± 0.013	0.230 ± 0.052	3	3	1.397 ± 0.091
	EC4158QH1	0.047 ± 0.005	0.082 ± 0.015	0.069 ± 0.011	1	1	0.092 ± 0.042
	EC4159QH2	0.049 ± 0.007	0.089 ± 0.013	0.055 ± 0.007	2	1	0.035 ± 0.025
	EC4160QH3	0.098 ± 0.015	0.034 ± 0.008	0.044 ± 0.008	2	3	2.257 ± 0.099
	EC3213QH34	0.036 ± 0.006	0.072 ± 0.007	0.171 ± 0.017	3	3	1.079 ± 0.046
	EC4161QH4	0.075 ± 0.012	0.060 ± 0.010	0.061 ± 0.009	2	1	0.050 ± 0.021
	EC4162QH5	0.001 ± 0.009	0.088 ± 0.020	1.021 ± 0.154	3	3	2.882 ± 0.255
	EC4163QH6	0.017 ± 0.003	0.135 ± 0.030	1.469 ± 0.304	2	3	1.848 ± 0.127
	EC4164QH7	0.053 ± 0.006	0.223 ± 0.025	0.245 ± 0.025	2	1	0.092 ± 0.034
	EC4165QH8	0.070 ± 0.008	0.127 ± 0.019	0.156 ± 0.026	1	1	0.065 ± 0.037
	EC4166QH9	0.061 ± 0.007	0.051 ± 0.010	0.070 ± 0.010	3	3	2.746 ± 0.163
pEHEC	EC801	0.040 ± 0.005	0.119 ± 0.017	0.121 ± 0.020	2	1	0.150 ± 0.042
	EC3983A	0.033 ± 0.006	0.057 ± 0.011	0.074 ± 0.010	2	1	0.081 ± 0.024
	EC3989A	0.063 ± 0.008	0.638 ± 0.036	1.402 ± 0.203	3	3	3.859 ± 0.138
aEPEC	EC3435A	0.044 ± 0.009	0.041 ± 0.007	0.060 ± 0.007	0	1	0.104 ± 0.064
	EC3457	0.110 ± 0.020	0.134 ± 0.023	0.135 ± 0.014	1	1	−0.001 ± 0.030
	EC3610A	0.019 ± 0.003	0.017 ± 0.004	0.033 ± 0.005	1	0	0.018 ± 0.023
	EC3727A	0.037 ± 0.005	0.034 ± 0.007	0.119 ± 0.021	0	1	0.268 ± 0.051
	EC3735A	0.046 ± 0.011	0.017 ± 0.011	0.108 ± 0.019	0	1	0.135 ± 0.027
	EC3768A	0.035 ± 0.012	0.078 ± 0.017	0.206 ± 0.028	0	1	0.128 ± 0.020
	EC4013A	0.048 ± 0.018	0.027 ± 0.011	0.101 ± 0.014	0	1	0.177 ± 0.032
	EC4039A	0.006 ± 0.003	0.060 ± 0.009	0.048 ± 0.007	1	0	−0.005 ± 0.013
NTEC	EC3536B	0.052 ± 0.005	0.224 ± 0.021	0.475 ± 0.034	3	3	0.966 ± 0.158
	EC3946A	0.052 ± 0.006	0.313 ± 0.056	0.404 ± 0.034	3	3	0.587 ± 0.127
Negative control	Lb broth	0.080 ± 0.0009	0.078 ± 0.001	0.071 ± 0.0004	0	0	

^a^Values are shown as mean of biofilm production ± standard error on polystyrene surfaces, SS: stainless steel, GS: glass slide at 25 °C. According to the biofilm

mass quantified with crystal violet staining assay at 570 nm isolates were labelled as the following: low, medium and thick biofilm formers

^b^Visible biofilms on stainless steel and glass slides and was scored as 0: no visible biofilm, scored on a scale from 1 to 3 to a thick biofilm at the air-liquid

^c^The presence and absence of visible pellicles biofilms was scored visually before staining with CV. Strains with visible pellicles at the air-liquid interface had OD_570_ ≥0.48
